# Socioeconomic Effects in Cyberbullying: Global Research Trends in the Educational Context

**DOI:** 10.3390/ijerph17124369

**Published:** 2020-06-18

**Authors:** Eloy López-Meneses, Esteban Vázquez-Cano, Mariana-Daniela González-Zamar, Emilio Abad-Segura

**Affiliations:** 1 Department of Education and Social Psychology, Pablo de Olavide University, 41013 Sevilla, Spain; elopmen@upo.es; 2 Research Institute in Social Sciences and Education, Vice-Rectory for Research and Postgraduate, University of Atacama, Copiapó 1530000, Chile; 3 Department of Didactics and School Organization, National University of Distance Education, 28040 Madrid, Spain; evazquez@edu.uned.es; 4 Department of Education, University of Almeria, 04120 Almeria, Spain; 5 Department of Economics and Business, University of Almeria, 04120 Almeria, Spain

**Keywords:** cyberbullying, health, socio-economic, education, peer relationships, scientific research

## Abstract

Nowadays, cyberbullying has become a problem of social harassment in numerous educational centres worldwide. New communication technologies have provided the perfect support for the development of this type of harassment in peer relationships, in addition to being linked to broad social and economic circumstances. In this study, the global trends of the socioeconomic implications of cyberbullying in the educational context have been analysed, from 2004 to 2019. Thus, a bibliometric analysis has been applied to 1128 articles, obtaining results of the evolution of scientific activity in this period. The articles are mainly associated with the thematic areas of Social Sciences, Psychology, Medicine and Computer Science. Seven main thematic axes have been detected, highlighting those related to the psychological aspect, adolescence, and the school environment. Likewise, the link of the main authors, institutions, and countries to these lines of research has been detected. The evidence has shown the interest and relevance of this topic at the international level. Future research lines propose different analyses about how certain socioeconomic factors influence this psychological harassment inside and outside the classroom.

## 1. Introduction

In recent decades, technological advances allow communication with other people through digital devices to be incorporated into our daily interaction dynamics. The current technological revolution has transformed the way we communicate and relate; and it has meant that, in the case of young people, school is not the only place where they live together, but a new framework has been introduced in which they primarily interact with other people, cyberspace [1,2].

Likewise, community life and the interpersonal relationships that are generated in the spaces become elements of relevance and development of the individual’s social competence. In the case of adolescents, most of the interactions take place in educational centres [3]. This is undoubtedly because the school is one of the main places where children and young people spend most of their time, and it is one of the spaces where their socialization takes place through daily coexistence. Therefore, this place is very important in the development of antisocial or criminal behaviours at school age and, also, it can be key in their prevention [4].

The quality of interpersonal relationships, in turn, affects the psychoevolutionary development of the students and it depends on them that they acquire an adequate development of social competence associated with cognitive and affective processes [5]. This competence has been considered from different theoretical perspectives, as the way to effectively carry out interpersonal relationships.

In the educational context, the importance of social competence to favour the social development of young people has been recognized [6,7]. The relevance of this theme is to improve the interpersonal relationships of individuals and prevent problems of violence between equals, such as harassment, and violence in the first couple relationships.

School violence has been nourished by ICTs to develop new violent dynamics, including cyberbullying. In this sense, ICT has revolutionised communicative, training and work processes [8]. This phenomenon is increasingly worrying because of its scope, since the population where it appears with the highest proportion is adolescents. It is one of the problems that cause the most concern in society, given the negative consequences that it entails, and which may be even more serious than those that appear when suffering from other types of abuse, such as child maltreatment by the family and from which the ideation of suicidal ideas and even suicide itself is not excluded [9,10].

In essence, cyberbullying (CB) is an indirect form of traditional harassment that shares the intrinsic characteristics of bullying, such as the aggressive act, intentionality, and repetition over time by one of several aggressors; although it has its own characteristics, such as anonymity, advertising on social medias and the difficulty of disconnecting from the cyber environment [11,12]. From this approach, CB is considered from the Triple Criminal Risk model in order to contribute to a broader definition of crime based on three sources of risk, that is, individuals, societies, and environments [13,14].

The purpose of this study is to analyse the socioeconomic implications on CB in the context of education, in order to examine the evolution of this crime in a global context determined by different economic and social factors.

The literature review has allowed finding documents on this topic, so the research question refers to determining if the evolution of scientific production is directly or indirectly associated with digital transformation. In other words, if scientific production in this area is linked to the development of platforms that promote research among academics, institutions or regions; And if in addition the development of social networks that encourages the development of CB is also linked, in some way, with the development of academic and scientific production.

The main objective of this study is to analyse global research trends on the socioeconomic aspects that affect cyberbullying in an educational context during the period 2004–2019.

Consequently, to obtain answers to the research question posed, a sample of 1128 articles from scientific journals selected from the Elsevier Scopus database was analysed. In this study, the bibliometric method has been applied to synthesize the knowledge base on global research trends regarding the socioeconomic aspects that have implications on CB.

The results obtained have shown the contributions in this field of research, so that it has allowed identifying the main driving agents, their current and future potential trends, in addition to revealing certain gaps in critical knowledge.

In this context, it is necessary to highlight that among the lines of research that are currently being developed in relation to the subject of the study, these refer, among others, to analysing the psychosocial impacts of cybervictimization and the barriers to seeking social support; to carry out socioeconomic analyses of the decision-making process that underlies the viewers who help the victims of CB; or to study the risk factors of CB and the association with perceived health in certain contexts and regions.

This study supposes an analysis of the scientific production and of the actors that stimulate the research on CB in an educational context, during the period 2004–2019, as well as the identification of the research lines and their evolution and transformation, assuming a contribution to decision making and reinforcing the relationship between science and technology.

It can be concluded that research in the socioeconomic aspects of CB in the field of education is a dynamic research topic, which evolves along thematic axes, and where it is observed that both researchers and institutions and countries are making efforts to study this aggressive behaviour that causes numerous problems in the health of adolescents.

This research has some limitations, which may be the basis for future research. They highlight the possibility of determining the relevance of authors, institutions, and countries, since in the scientific field there is a paradox that some publish few articles, but they are very relevant in a certain subject area. Therefore, to overcome these limitations, in the research work, the bibliometric analysis must be completed with other qualitative and quantitative methodologies. On the other hand, the study could be expanded with publications compiled in other bases of data.

In order to achieve the stated objective, this research work has been structured as follows: Section 2 details the scope of the study and justifies the relevance of this research topic, delimiting the unit of analysis and providing a theoretical framework that acts as a guide. Section 3 presents the methodology applied in this research and the process of selecting data from the sample of scientific contributions. Section 4 shows the main results and their discussion. Finally, Section 5 presents the conclusions obtained and the main future research lines.

## 2. Research Scope

This section is the result of the previous analysis and review of the literature, while its purpose is to act as a guide and framework in the global investigation of socioeconomic aspects with implications for CB in the educational context. Thereby, firstly, the backgrounds of this topic is described, and then, the theoretical model and a series of interrelated terms are incorporated that allow the study in the field of knowledge to be conceptualized, and the aim of this research to be consolidated.

### 2.1. Backgrounds

International research on bullying and harassment among schoolchildren has fundamentally gone through four time periods [15,16]. From 1970 to 1980, its systematic study began, with the Norwegian researcher Olweus pioneering this analysis in his book *Aggression in School: Bullies and Whipping Boys* [17]. Later, in the 1980s, intervention programs were developed after the previous investigation, such as the *Olweus Prevention Bullying Program* [18]. From 1990 to mid-2004, studies on bullying became international [19] and, from 2004, it is when the term cyberbullying is added to refer to cyberspace as a new medium in which assaults occur [20].

Most of the interactions of adolescents occur in educational centres. This is undoubtedly because the school is one of the main places where children and young people spend most of their time, and it is one of the spaces where their socialization takes place through daily coexistence. Therefore, this place is important in the development of antisocial or criminal behaviours at school age and, also, it can be key in their prevention [21]. On the other hand, the current technological revolution has transformed the way of communication and relationship, and this has meant that, in the case of young people, school is not the only place where they coexist, but a new framework has been introduced in which they primarily interact with other people, cyberspace, which is, fundamentally, a place of communication [22].

Aggressive behaviours that occur in virtual space seem to have very serious consequences for victims. Specifically, these consequences for young people are the devaluation of their self-esteem, their self-confidence, academic problems, poor interpersonal relationships, and poor psychosocial adjustment. These negative effects can be as severe as those that occur from traditional abuse and harassment [23,24].

A review of the literature on the research topic has established a framework to unite the theoretical basis and terminology of the global study of the socio-economic implications of CB in the educational environment. For this reason, Table 1 shows the main documents examined to focus on the theoretical and conceptual structure of the research topic. This examination has allowed determining the problem, the purpose and the objective of the investigation, as well as obtaining the key terms to apply the methodology specified in the following Section 3.

### 2.2. Framework

From the literature review related to the different theories consulted in this research, the framework for the socioeconomic implications of CB in the educational system is established. This selected theoretical model supports the CB argument as a problem of the digital age in the educational environment.

In this context, the Triple Criminal Risk (TCR) model is configured as an integrative theory or meta-theory of crime. In other words, this criminological model assumes, as an axiom, that it is probable that there is not a single criminal genesis process [40]. Criminal behaviour would become more likely from the reciprocal interaction between various factors, that is, from the sources of risk. Likewise, this model is situated on the criminological side, called Developmental Criminology. This criminological theory has unified all possible pro-criminal influences into the categories: Personal Risks (PR), Gaps in Prosocial Support (GPS) and exposure to Criminal Opportunities (CO) [41,42].

There are also previous researches of CB behavior based on general criminological theories. Three of these theories have a greater theoretical incidence on personal factors: the Theory of Tension, by Robert Agnew; the Social Cognitive Theory with its mechanisms of moral disassociation, by Albert Bandura; and Ajzen’s Theory of Reasoned Action; and a fourth that affects aspects of opportunity: the Theory of Daily Activities [43,44,45].

The advantage offered by studying the phenomenon of CB taking into account the TCR model, is because it is a more comprehensive theory that integrates internal and external elements of the individual. Furthermore, it attempts to contribute to a more general definition of crime based on three sources of risk: individuals, societies, and contexts [46].

In this context, research requires defining the main terms conceptually, with the fundamental purpose of contextualizing their intrinsic circumstances. Firstly, bullying is defined as any form of psychological, verbal or physical abuse produced between schoolchildren, repeatedly over a certain period of time. In the same way, it could be said that it is a type of conduct directed to do damage, repeated over time and that occurs in the environment of an interpersonal relationship characterized by an imbalance of power.

On the other hand, when social networks are used as a means of aggression, this phenomenon is called CB. One of the most frequent manifestations of this is the publication of photographs, which can cause annoyance to its protagonists, so that their contacts see the images. Also, very frequent the creation of Web pages or groups destined to attack, circumvent or denounce some intimate aspect of the victim [47,48].

CB has a differentiating element through the use of technology, which is why it is defined as the repeated and intentional damage caused through electronic means, such as mobile or the Internet, and carried out by an individual or a group against which the victim cannot defend herself/himself [49,50].

Moving forward in its conceptual definition, the CB is about an intentional aggression, by a subject or a group, with the intervention of electronic forms of contact; and that the person to whom it is addressed cannot defend itself of it. CB is also considered to consist of cruel and hostile conduct towards another person through the sending or publication of harmful material or involvement in other forms of social aggression using the Internet or other digital technologies. Finally, it is also considered socially inappropriate online behaviour: harassment, humiliation, intimidation, insults or threats in messages, taunts and use of inappropriate language.

Educational policies and didactic and pedagogical trends increasingly advocate the use of technology inside and outside the classroom as a support mechanism for teaching and learning [51]. Nowadays, schoolchildren, unlike their parents, are digital natives. These new generations have a greater knowledge of technology than their parents, these being, at best, digital immigrants. For this reason, schoolchildren find a haven of privacy online where they can interact with their peers. In this communication, schoolchildren find a refuge that when assaulted leaves them in a devastating state of vulnerability [52]. Hence, for harassers, the digital environment is a perfect and effective means to attack their victims, since the technologies do not pose any difficulties to them, they avoid face-to-face confrontation, and the scope of the damage they achieve is considerable in proportion to the action they execute. Our lives are digitized and digital, which requires a properly trained citizenry to face the challenges and novelties that have arisen [53].

Regarding the psychological sequelae that victims are likely to suffer, it has been observed that they usually occur during adolescence and throughout their adult life, with symptoms such as high levels of anxiety, depression-like mood disorders, helplessness, low self-esteem, degradation of their self-concept, or different types of psychosocial maladjustments [54]. It has also been observed that numerous victims, while they are suffering bullying, present low academic performance, difficulty concentrating and truancy. In the future they will be more vulnerable to stress, negative emotions such as fear and having suicidal thoughts [55,56].

Consequently, CB differs mainly from bullying in: (i) a greater scope, since with a single gesture the harasser can spread a hoax to thousands of people online; (ii) the impossibility of escape, the victim has no way of avoiding the harassment situation; (iii) the anonymity of the harasser; and (iv) the permanence of the harassment, the hoax poured in cyberspace can be difficult to eliminate or perpetuate over time, so that the victims will assume a constant re-experience of the harassment experience. All this, in addition to the fact that the harassment scheme, due to its playful nature, transmits to the harasser a low sense of guilt and a high sense of pleasure. Table 2 lists these two forms of crime, bullying [57] and CB [58,59], through their typologies.

Among the main differences that are made explicit in CB, they highlight: (i) intentionality, the aggressor must have the intention of harming the victim so that a true phenomenon of harassment occurs; (ii) repetition, CB, like bullying, requires that the aggression be reproduced more than once, (iii) the imbalance of power, which can result both from the victim’s helplessness in the face of aggression and from technological anonymity, since the person cannot do anything against the aggression, cannot delete a photo or video from the Internet that has already been broadcast; (iv) absence of physical and social feedback between the participants, that is, as there is no physical contact between the aggressor and the victim, this means that it is not possible to know the victim’s reaction, but it does promote disruptive, uninhibited behaviour in the victim aggressive and impulsive; and (v) open channel, unlike bullying, in which harassment of the victim occurs in a specific space (school, institute or on the way to/from home), in CB, with the possibilities of new technologies, aggressor can commit his attacks at any time [60,61,62,63,64].

Another term related to the research topic is adolescence, defined by the World Health Organization (WHO) as the period of human growth and development that occurs after childhood and before adulthood, that is, between 10 and 19 years old [65]. This is one of the most important transitional stages in a person’s life, characterized by an accelerated rate of growth and change, and conditioned by various biological processes [66,67].

Furthermore, the crime of CB occurs in the context of the digital environment, mainly social networks [68,69]. These are Internet sites made up of groups of people with common interests or activities (friendship, kinship, work), with the objective of communicating and exchanging information. They emerged in 1995 with the creation of the website classmates.com by the American, R. Conrads, to bring together former classmates from college or university.

As for the socioeconomic aspects that influence CB, socioeconomics is an alternative economic and social paradigm to neoclassical economics programmatically proposed by the German sociologist Etzioni (founder, in 1988, of the World Society for Socioeconomics) in his work *The Moral Dimension of Economy* [70,71]. In this line, the socioeconomic level of a person or a group includes a series of economic, sociological, educational and labour variables for which it is classified within a social hierarchy [72,73].

Also, the socioeconomic study allows to know the economic, social, cultural and work environment of a person; although in the study of the socioeconomic factors that have implications in CB, it is key to determine how the socioeconomic level affects education [74,75].

Hence, factors such as: (i) the income level of the family unit stand out, that is, the economic situation of the family can help and hinder the obtaining of education and this be associated with aggressive, undisciplined children, they lack affection and with low self-esteem; (ii) the educational level of the parents, since the lack of education of the parents can suppose a negative attitude towards education, and hinder the child academically; (iii) gender, the availability of education for girls varies by country and culture, so that in some the content of education is limited in certain specific areas; (iv) race, in some countries, for example, indigenous and Afro-descendant populations are limited to access to education; (v) the social environment, that is, factors such as where they live, the circle of friends and family life determine school performance and the educational level they achieve; and, lastly, (vi) the state budget for the education sector may have an impact on the creation of programs to sensitize the educational community to CB [76,77,78,79].

## 3. Materials and Methods

### 3.1. Bibliometric Method

Bibliometrics studies the main characteristics and evolution of a scientific discipline through its publications, mainly from its computation and from the analysis of relational indicators. This science uses bibliometric indicators to measure the existing information on the results of scientific activity in any of its documents. This set of indicators allows to quantitatively express the bibliographic characteristics of scientific publications. A bibliometric indicator synthesizes a certain bibliographic characteristic or a combination of these using a numerical value, which denotes interest when it can be compared with observations from other document sets, from other geographic areas, institutions, disciplines, specific areas of knowledge, or databases, and thus study its evolution over time [80,81,82].

The objective of this research work is to analyse global research trends in socioeconomic factors that have an impact on CB at the educational level. To achieve this, a quantitative analysis has been performed, using the bibliometric method. This is a documentary method that has reached an important development in the last decades and has allowed to review the scientific knowledge of different publications in many scientific areas [83,84,85,86].

The methodology applied in this study has been the search for scientific publications related to the object of study in the Scopus database, chosen for its outstanding coverage and reliability in the peer review, as has been used in other analyses applied with success [87,88,89].

Bibliometric techniques are the basis for measuring the effort and impact of scientific activity, since, for example, the number of scientific documents published in a country can be quantified, both by an institution and by a researcher or team of researchers. The objective of bibliometrics is, therefore, to study science based on the analysis of scientific publications published in a certain territory or field of knowledge.

### 3.2. Data Collection

Based on the revised literature of the study topic, mainly in Table 1, the terms chosen in the search string are “cyberbullying”, “cyber-bullying”, “social”, “economic” and “education”, joined by Boolean connectors.

**Figure 1:**
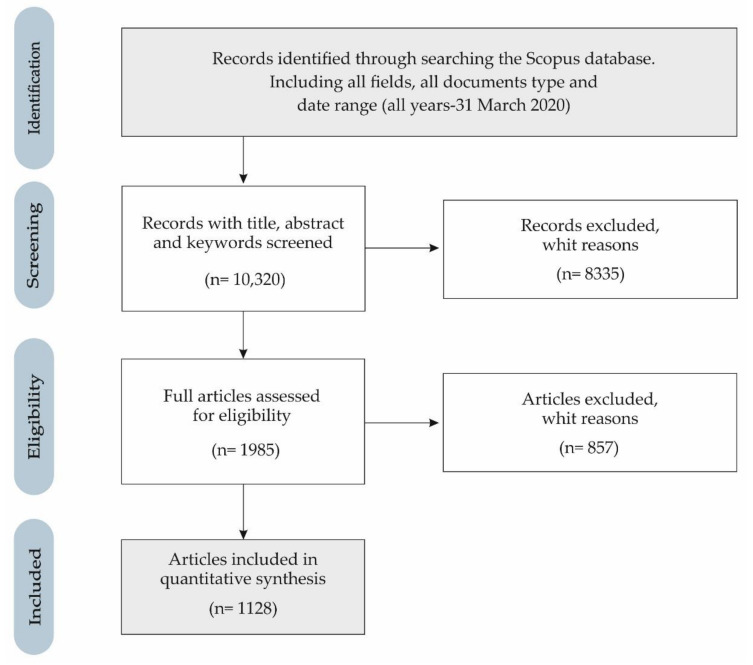
PRISMA flowchart of the applied methodology.

The process followed in the selection of the sample conforms to the flow chart of Figure 1, according to the Preferred Reporting Items for Systematic Reviews and Meta-Analyses (PRISMA) guidelines [90]. Thereby, in phase 1 (identification) 10,320 records were identified from the Scopus database, considering all the fields for each of the search terms (cyberbullying, cyber-bullying, social, economic, education), all types of documents and all the data in the data range (all years—31 March 2020). In the next phase 2 (screening) the option of “article title, abstract and keywords” is chosen in the field of each term, so that 8335 records are excluded. Subsequently, in phase 3 (eligibility), with 1985 records, the data range is chosen from all years to the last full year (2019), and the document type only selects the articles, for quality assurance that they have derived from the peer review process. Hence, this phase excluded 857 records. For these reasons, in the last phase (included), the final sample is collected with 1128 articles. Essentially, the search selected records from the subfields title, abstract and keywords, in the period that contains the publication of the first article on the research topic (2004) until the last full year (2019), that is, a period of 16 years has been analysed, in the same way that it has been applied successfully in various works that have used bibliometric methodology [91,92,93]. The representation of this sample of articles is supported by the proven quality of the Scopus database, regarding the indexing protocol, in addition to the systematic procedures of the search criteria.

### 3.3. Data Processing

The bibliometric methodology has been developed to analyse the scientific communities associated with this research topic. The relationships between the main authors, affiliations, and countries in global research on CB have been analysed, explained through the co-authorship of each article, and the number of citations received has also been considered. A publication by the rest of the scientific community, which quantifies the impact achieved by this publication. In addition, the relationships between the keywords of all scientific articles on the research topic, based on co-occurrence, have been analysed [94,95,96].

In the methodology used, the co-authorship analysis allows observing the articles with citations and cited references, which can show scientific knowledge and trends in each discipline in order to establish research strategies. Accordingly, bibliometric instruments are used in evaluation operations. The authors, institutions and the countries are determined from the citations shared by the other elements, which are relevant to the discipline [97,98]. This allows generators of scientific production (authors, institutions, and countries) to be substitutes for the ideas they represent when grouping [99].

Likewise, the co-occurrence analysis is used in order to provide a graphic visualization of the connection of the key terms of the analysed documents. The co-occurrence networks provide a graphic map of the relationships between the authors, institutions, countries, or keywords of a sample article on a given research topic. In this sense, the proximity relationship of two or more keywords is established in a text unit. Thus, if the keywords coexist in a sentence, that is, they appear together, there is a probability of semantic relationship [100].

The co-occurrence criteria group strongly related keywords in the set of articles in the sample. This analysis examines the articles to search for two or more keywords that tend to be together [101]. It is established that if there are concurrent concepts, a category is generated. In this way, two or more keywords will be co-occurring if they frequently appear together in a sample of articles and if, occasionally, they are separated in the other articles.

In summary, indicators of collaborative structure measure the relationships between authors, institutions, and countries that contribute to a research topic over a period. In this study, these indicators have been analysed with network processing tools and maps, due to their proven reliability and convenience in bibliometric methodology [102,103].

To obtain these co-authorship and co-occurrence maps, the online software tool VOSviewer, (version 1.6.15, Leiden University, Leiden, The Netherlands) was used in this study. The VOSviewer tool (Visualizing scientific landscapes) offers reliability and guarantee in bibliometric methodology, as a tool for mapping and processing article data, since it has been used in other bibliometric studies. This tool allows the analysis of co-citation and co-occurrence, to visualize relationship maps and network links between authors, institutions, countries, and keywords. Subsequently, this software tool allows recognizing research trends based on the use of keywords in research articles [104,105].

Finally, all the results obtained allow the evaluation of research and scientific activity in the CB research area. Likewise, it is necessary to recognize that Bibliometric instruments allow the analysis and description of innovation processes, to identify themes, authors, institutions and countries, in addition to monitoring interactions between them. In this sense, the content, and options for success of innovation in a research area will depend on the intensity of these relationships and links. The results obtained from the application of the bibliometric method to the sample are revealed to be useful for researchers, academics, and decision-making to optimize for the rest of the agents involved in this research topic.

## 4. Results and Discussion

Section 4 presents and discusses the results of the global evolution of scientific production and activity in the socio-economic implications of CB in the educational context. Likewise, the findings about the distribution of publications by subject area and by journal are presented. These results together with the analysis of the main keywords associated with the most productive authors, institutions and countries worldwide, and with the analysis of the main associated keywords, allow us to determine the current and future trends of this important research topic at the international level.

### 4.1. Scientific Production

**Figure 2:**
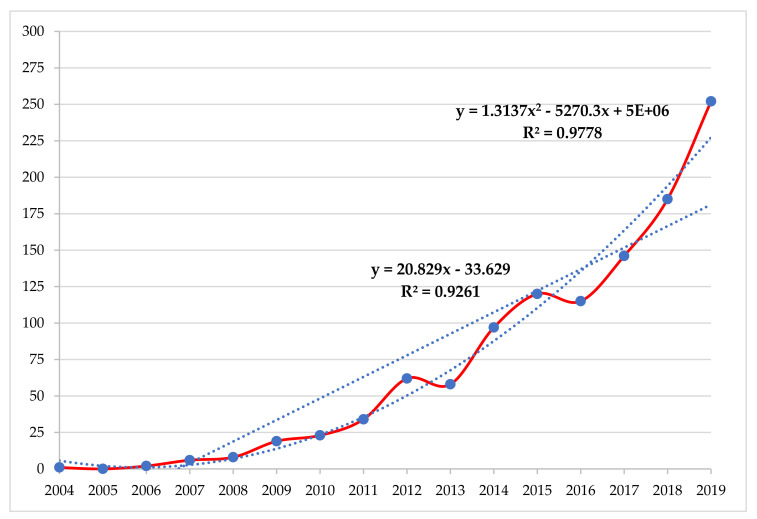
Evolution of Scientific Production (2004–2019).

Figure 2 shows the progress of the total articles in the sample that have been identified in the search carried out in the Scopus database. The polynomial trend line of order 2 illustrates the growing relationship between the number of articles published on the socioeconomic aspects of CB in the educational environment throughout the time horizon examined, since the data change direction in this period. Thereby, the curve represents shows its goodness with an R^2^ of 0.9778. On the other hand, the linear trend line shows that scientific production on the research topic has increased at a constant rate from the end of 2006 to 2019, that is, over a period of more than twelve years. It is observed that the value of R^2^ is 0.9261, which is a good fit of the line to the data set. In this sense, both trend lines show their goodness of fit since their R^2^ value is close to 1.

Furthermore, this result also allows us to observe that during the last triennium (2017–2019) 51.58% (583) of the total articles have been published, that is, in the last three years more than half of the total documents on this research topic, which indicates the special interest and relevance it arouses in the academic and scientific community at the international level [106].

Furthermore, this result is confirmed if it is observed that in the last five years (2015–2019) 818 articles have been published, which represent 72.52% of the total; while in the last decade (2010–2019) 96.81% (1092) of total contributions were published. On the other hand, in the first year analysed (2004), only one article was published; while in the last year studied (2019), 252 articles (22.83%) were published.

Table 3 display the ten most cited documents on the research topic of the socioeconomic factors associated with CB globally, during the period 2004–2019. It is observed that the article with the most citations date from 2008 (1316), and it is about the nature and impact of CB on the pupils of the secondary school [107].

The most cited article is Cyberbullying: Its nature and impact in secondary school pupils [107] by Smith (affiliated with Goldsmiths, University of London, and Unit for School and Family Studies, Goldsmiths College, New Cross, both in London, UK) and Mahdavi et al. (affiliated with Goldsmiths, University of London, London, UK). This document has 1322 citations in the Scopus database, ranked first in the Medicine category, followed by Psychology. In relation to the thematic areas in which the ten most cited articles are associated, 50% are classified in the Medicine category, 30% in Arts and Humanities, and 20% in Social Sciences.

Regarding the distribution of articles by language, most of this research topic is written in English (88.86%), since it increases their audience, as it happens mainly in searches made in the Scopus database [117]. Articles have also been published in other languages, such as Spanish (6.26%) or French (1.11%), while the rest do not exceed 1% of total contributions.

### 4.2. Subject Areas and Journals

In the time horizon examined, articles related to CB research are grouped into different areas of knowledge. Hence, the sample of 1128 articles is classified into 23 subject areas, according to the Scopus database. In this sense, it should be noted that an article could be classified in more than one subject area, depending on the interest of the author or authors of the article and the editor of the journal.

**Figure 3:**
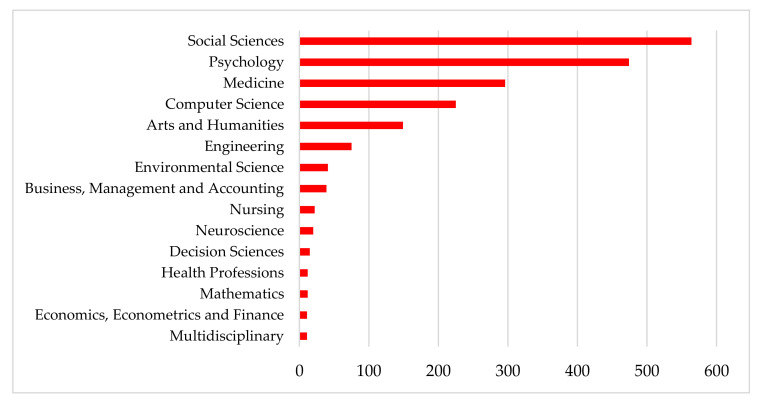
Main Subject Areas (2004–2019).

Figure 3 presents the classification by subject areas of the articles in the global research on CB, during the period 2004–2019. Social Sciences is the category that collects the most articles with 28.51% of the articles published (564) in the total sample. Next, it follows the Psychology (23.71%, 474), Medicine (14.81%, 296), Computer Science (11.26%, 225), Arts and Humanities (7.45%, 149), Engineering (3.75%, 75) and Environmental Science (2.05%, 41). The five most relevant thematic areas group 85.44% of the articles published in the period 2004–2019. The rest of the thematic areas do not reach 2% of published works.

Most of the articles on CB are associated with the thematic area of Social Sciences, which is made up of various disciplines such as education, law, economics, history, sociology and communication [118]. When considering the socioeconomic aspects of the CB, it is relevant the first position that it occupies within the ranking of the 15 thematic areas that carry out the greatest scientific production.

As can be seen in Figure 3, when considering CB as a social problem, psychology comes second. This discipline that attends to the study of individuals as social beings and their behaviours, recognizes in the CB the presence of psychological consequences in its victims. Some of the alterations observed by various authors coincide in the loss of self-esteem, mood disorders such as depression, high levels of anxiety, among others [119].

For its part, Medicine as a science that is at the service of the individual, seeks among its objectives to achieve the prevention of diseases and the promotion and preservation of health. In this way, understanding the CB as a problem that worries not only the relatives and surroundings of the victims, but also the health professionals, due to its powerful growth in cases in recent years and for acting as a bridge to other actions, such as rapes, suicides, addictions and another series of physical and psychological abuse [120].

As seen, CB especially affects adolescents, being a virtual type of bullying. New technologies and sometimes the misuse of them, have led to this type of harassment has seen the percentages of those affected shoot up. Thus, the relevance of the topic at present and the importance of prevention are two of the issues in which the research coincides [121]. This situation is demonstrated in the growing volume of research and that the Computer Science area appears in a relevant fourth place.

Finally, the results demonstrate a significant association between certain risk factors and the possibilities that the CB phenomenon will spread among young people. Among other social factors, the intensive use of the Internet, membership of social networks, an erroneous perception of privacy on the Internet and from the economic point of view, greater economic solvency, both in the role of victim and that of stalker, were some of CB risk factors [122,123].

In this way, it is confirmed that the socioeconomic aspects intervene and impact the CB due to the social nature of the phenomenon, thus being the most cited articles and, to a certain extent, most relevant to the scientific and academic community, those that collect contributions associated with the Social Sciences [124].

**Figure 4:**
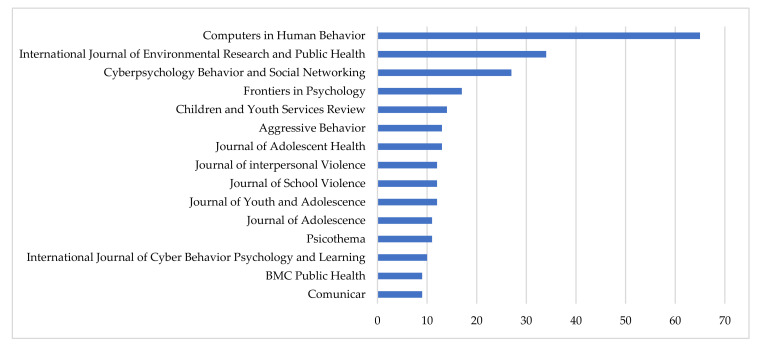
Main Journals (2004–2019).

Figure 4 presents the fifteen most productive scientific journals on this research topic. Taking into account the number of articles published and the percentage they represent of the total sample, this ranking is led by *Computers in Human Behavior* (65, 5.76%) and *International Journal of Environmental Research and Public Health* (34, 3.01%). They follow them, in order, *Cyberpsychology Behavior and Social Networking* (27, 2.39%), *Frontiers in Psychology* (17, 1.51%), *Children and Youth Services Review* (14, 1.24%), *Aggressive Behavior* (13, 1.15%), *Journal of Adolescent Health* (13, 1.15%), *Journal of Interpersonal Violence* (12, 1.06%), *Journal of School Violence* (12, 1.06%) and *Journal of Youth and Adolescence* (12, 1.06%). The rest of the journals in this Figure 4 do not reach 1% of the total contributions.

Among the most productive journals on this research topic, the second position of the *International Journal of Environmental Research and Public Health* (*IJERPH*) is remarkable, with 23 articles published in 2019, which represents 67.65% of the total on this topic, and, furthermore, all are classified in the thematic areas of Environmental Science and Medicine [125,126].

### 4.3. Analysis of Keywords

The top twenty keywords associated with the total sample of 1128 articles on the global research of socioeconomic factors linked to CB in the educational context, according to the Scopus database, are “adolescent”, “Internet”, “child”, “psychology”, “social media”, “major clinical study”, “crime victim”, “computer crime”, “victim”, “adolescent behavior”, “adolescence”, “aggression”, “social networking (online)”, “school”, “education”, “mental health”, “depression”, “victimization”, “peer group” and “sex difference”.

**Figure 5:**
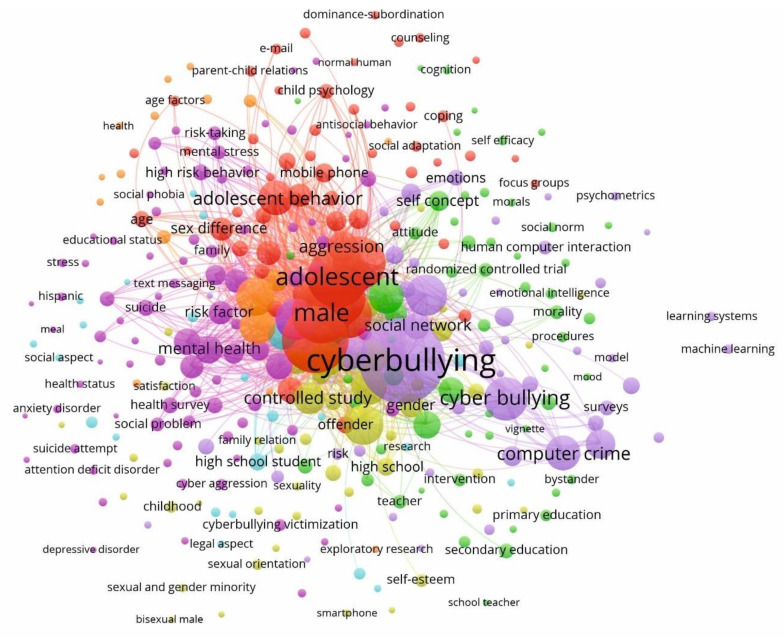
Keywords network based on co-occurrence analysis (2004–2019).

Figure 5 shows the keyword network on this research topic, based on co-occurrence analysis, from the VOSviewer software tool. One of the main utilities of the bibliometric method is the examination of the analysis units of the article text, that is, of the keywords extracted from the title and abstract fields [127]. Therefore, seven clusters of keywords composed of interrelated units have been detected, on the topic of socioeconomics aspects on CB in an educational context at a global level.

The pink cluster, the most numerous, groups 26.33% of the keywords in the articles. The main keyword is “psychology”, with 159 co-occurrences. This keyword is associated with “academic achievement”, “addiction”, “aggressiveness”, “alcohol consumption”, “anonymity”, “antisocial behavior”, “anxiety”, “anxiety disorder”, “attention deficit disorder”, “autism”, “avoidance behavior”, “awareness” “behavior addictive”, “body image”, “bully”, “chat room”, “communication”, “comorbidity”, “coping behavior”, “cyber aggression”, “cyber harassment”, “cyber victimization”, “cyberbullying perpetration”, “cyberbullying victimization”, “depression”, “depression disorder”, “disease association”, “distress syndrome”, “drug dependence”, “educational status”, “emotion”, “emotionally”, “family”, “fear”, “feeding behavior”, “health status”, “health survey”, “high risk behavior”, “Hispanic”, “impulsiveness”, “insomnia”, “intelligent quotient”, “internet addiction”, “interpersonal communication”, “life satisfaction”, “loneliness”, “meal”, “mental disease”, “mental disorder”, “mental health”, “mental stress”, “middle school student”, “observational study”, “parental mediation”, “physiology”, “physical activity”, “predictive value”, “prevalence”, “problematic internet use”, “psychological distress”, “psychological theory”, “psychological well-being”, “psychosis”, “suicide attempt”, “technology”, “text messaging”, “utilization”, “wellbeing”, or “young population”.

The green group (17.16%) is headed by “student” (116) and it is associated with “adolescence”, “adolescent health”, “attitude”, “behavior”, “behavior change”, “bystander”, “cell phone”, “clinical article”, “cognition”, “college student”, “cooperation”, “education”, “emotional intelligence”, “empowerment”, “extraversion”, “helping behavior”, “human issue”, “intervention”, “mood”, “moral disengagement”, “morality”, “moral”, “neurosis”, “organization”, “organization and management”, “parents”, “perception”, “physical violence”, “prevention and control”, “primary education”, “primary school”, “program evaluation”, “psychological model”, “quality of life”, “responsibility”, “school health service”, “school teacher”, “secondary education”, “self-concept”, “self-efficacy”, “social behavior”, “social environment”, “social norm”, “teacher”, “teaching”, “university”, “university student”, or “vignette”.

The red group (16.57%) led by “adolescent” (340) and it is associated, among others, with “adaptative behavior”, “adolescent behavior”, “adolescent psychology”, “age”, “age factors”, “aggression”, “anger”, “bullying”, “cellular phone”, “child”, “child behavior”, “child parent relation”, “child psychology”, “coping”, “counselling”, “culture”, “dominance-subordination”, “e-mail”, “focus groups”, “information technology”, “Internet”, “interpersonal relations”, “juvenile”, “middle aged”, “mobile phone”, “offender”, “parent-child relations”, “parenting”, “peer group”, “psychological aspect”, “risk assessment”, “school child”, “sex difference”, “sex distribution”, “social adaptation”, “social dominance”, “social perception”, “social psychology”, “software”, “victimization”, or “ videorecording”.

The yellow group (13.91%) is headed by “victim” (116) and is associated with “adulthood”, “bisexuality”, “childhood”, “cybervictimization”, “decision making”, “empathy”, “family relation”, “gender”, “girl”, “harassment”, “heterosexuality”, “high school”, “homophobia”, “major clinical study”, “middle school”, “minority group”, “physical abuse”, “prevention”, “protection”, “race”, “self-control”, “self-esteem”, “sexual and gender minority”, “sexual behavior”, “sexual harassment”, “sexual orientation”, “sexuality”, “skill”, “smartphone”, “social competence”, “social skills”, “verbal hostility”, “young adult”.

The violet cluster (13.91%) is led by “cyberbullying” (666) and is associated with “children”, “computer crime”, “cyber bullying”, “cyber-bullying”, “cyber-victimization”, “cybercrime”, “emotions”, “Facebook”, “friendship”, “human computer interaction”, “learning”, “machine learning”, “model”, “obesity”, “online communication”, “online harassment”, “parent”, “peer victimization”, “physician”, “privacy”, “psychosocial disorder”, “reliability”, “risk”, “school bullying”, “sexting”, “social media”, “social network”, “social networking (online)”, “traditional bullying”, “twitter”, or “youth”.

The blue cluster (6.80%) is headed by “school” (78) and is linked with “behavior disorder”, “bullying victimization”, “child safety”, “computer”, “cultural factor”, “emotional disorder”, “ethnicity”, “gender identity”, “high school student”, “law”, “legal aspect”, “outcome assessment”, “safety”, or “public health”.

Finally, the orange cluster, the least numerous (5.33%), is led by the keyword “crime victim” (138) and is linked with other terms, such as “automutilation”, “child abuse”, “epidemiology”, “friend”, “health”, “self-injurious behavior”, “socioeconomic factors”, “socioeconomics”, “violence”, or “well-being”.

Consequently, from the clusters formed in Figure 5, seven different lines of research can be inferred in relation to the socioeconomic aspects that influence CB in the educational context. The first research line is linked to the term psychology and related to the thematic axis of CB. This line focuses on the study of the profile of the aggressor (addiction, aggressiveness, anonymity, antisocial behaviour, or feeding behaviour), of the victim (anxiety, body image, fear, family, education status, emotion, or psychological well-being), of diseases and associated disorders (comorbidity, anxiety, mental disease, mental disorder, mental health, mental stress, disease association, or distress syndrome), or the analysis of this type of aggression (coping behaviour, cyber aggression, cyber harassment, cyber victimization, cyberbullying perpetration, cyberbullying victimization, or drug dependency). This thematic axis in studies the significant impact that CB has on the psychosocial adjustment of adolescents, which increases depressive symptoms and the problematic use of the Internet and social networks [128,129].

The second line associated with the term student, is related to the study, mainly, of the influence of CB on academic self-esteem and school perception (college student, education, organization, organization and management, school health service, school teacher, primary school, or secondary education). Although psychosociological factors associated with this aggression are also linked to this line in analysis (attitude, behaviour, behaviour change, cognition, extraversion, helping behaviour, or psychological model). This line is widely studied since CB was considered as a new form of educational bullying [130].

This third line of research is associated with the term adolescent, so it fundamentally studies the characteristics of this period, that is, physical growth and psychological development, as well as how and why it is so directly associated with CB (adolescent behaviour, adolescent psychology, juvenile, middle aged, age, age factors, or peer group) [131]. This thematic axis is also associated with the use of technologies, such as digital natives (e-mail, software, videorecording, information technology, Internet), and close family relationships at this vital stage (child parent relation, parent-child relations or parenting) [132].

The fourth axis is linked to the term victim and, specifically, is devoted to examining the characteristics that a vulnerable person makes of the aggressor (empathy, self-esteem or childhood), and with sexuality itself (gender, bisexuality, heterosexuality, sexuality, sexual orientation), sexual and gender minority, or sexual behaviour). This line has also been studied in recent years, due to the staggering of sexually aggressive or coercive behaviour facilitated using electronic devices [133].

The fifth axis is associated with the main term of the study, that is, CB. This term, as the central axis of the research, is the one with the most co-occurrences. This line is linked in a general way with all aspects of the study of this type of aggression, and mainly with its digital environment [CB social media] (computer crime, Facebook, human computer interaction, online communication, online harassment, social media, social network, social networking (online), or twitter).

This sixth line is associated with the term school in the context of CB. This axis is directly associated with the studio of the second line (student). Here, mainly, the aspects of the educational centre are examined as a space where this type of aggression begins [134] (bullying victimization, child safety, computer, cultural factor, ethnicity, or high school student).

Finally, the sixth line is associated with the term crime victim and studies both from the victim’s point of view (automutilation, child abuse, epidemiology, or self-injurious behaviour) [135] and, more recently, the social and economic factors that influence in the development of CB (socioeconomic factors, socioeconomics) [136].

On the other hand, Table 4 displays the twenty main keywords by number of occurrences and the total of link strength. As can be seen, each keyword is associated with one of the clusters in Figure 5. 

In this ranking the most represented clusters are, with five keywords each, red (adolescent, Internet, child, adolescent behaviour, aggression) and violet (cyberbullying, cyber bullying, social media, computer crime, social networking (online)). Also, the occurrences attribute indicates the number of documents in which a keyword appears, while the total strength of the link indicates the number of publications in which two keywords appear together. Also, the occurrences attribute indicates the number of documents in which a keyword appears, while the total link strength indicates the number of publications in which two keywords appear together. Consequently, the keyword “cyberbullying” is the one that has more occurrences (666) and greater total link strength (1929). In relation to the highest number of occurrences, it is followed by “bullying” (408), and in terms of the total link strength, it is followed by “adolescent” (1899).

**Figure 6:**
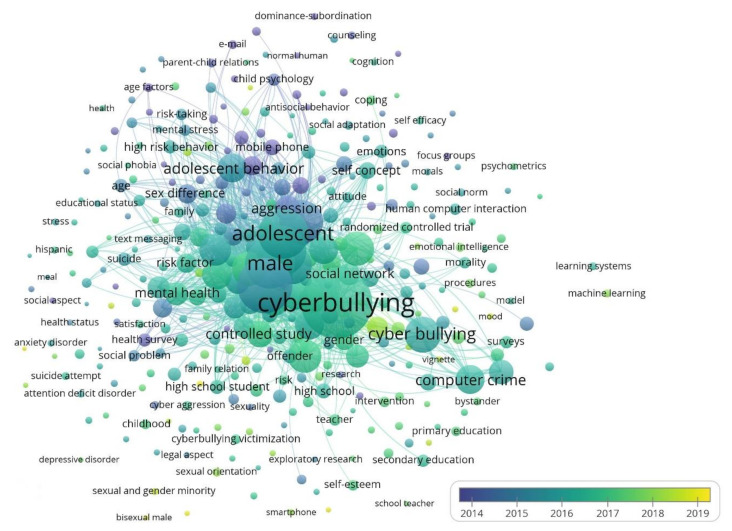
Evolution of keywords network based on co-occurrence analysis (2004–2019).

Figure 6 shows the evolution of each group of keywords during the period examined, 2004–2019. This Figure represents the value over time of the keywords associated with the different research topics, depending on when they arose and were linked to the contributions on socioeconomic aspects with implications on CB in the educational environment. The terms that were first linked to this topic (2004–2014), as terms of reference, have had a notable influence on those that have subsequently emerged. Next, a second period (2014–2016), a third period (2016–2018), and a fourth period (2018–2019) can be identified. In this way, it is observed that the research topics related to psychology, student, adolescent, victim, and school have been the most studied during the time horizon, mainly, from 2004 to 2018. In the same way, it happens with the line of the CB that, as a central theme, it continues to evolve during the period analysed. The topic related to crime victim is the most recent and the one that is contributing new terms to the study, so since 2018 researchers are dedicating their efforts to analyse the social and economic aspects that have a direct influence on this type of virtual harassment, and, in this sense, they are in the process of generating new lines of research.

In this context, new keywords are emerging or being given a new application, such as “policing”, “blackmail”, “teenage slang”, “expletives”, “truancy”; “trolling”, “tokenization”, “technoself”, “technoference”, “stuttering”, “stalking”, or “speedbump”. Also, this study is associating theories, such as “social theory” or “criminological theory” [137,138].

Another series of keywords are associated with the works that study the social and economic aspects in the CB, among these are “patient counselling”, “social stigma”, “ethnic minority”, “ethnic difference”, “minority groups”, “gender based violence”, “gender nonconforming”, “gender disparity”, “cyber homophobic bullying”, “positive digital citizenship”, “management system information”, “absenteeism”, “school absenteeism”, “deafness”, “hearing impairment”, “sociocultural”, “social skills”, “social phobia”, “economic status”, “developing countries”, “global health”, or “socioeconomically disadvantages”.

### 4.4. Authors, Affiliations and Countries

Table 5 shows the main characteristics of the 10 most prolific authors in this research topic. The total sample of articles has been written by 2674 authors. The five most productive authors were Vandebosch (Universiteit Antwerpen), Ortega-Ruiz (University of Cordoba), Wright (Masaryk University), Van Cleemput (Universiteit Antwerpen) and Casas (University of Cordoba). Eight authors of this ranking are of European origin, highlighting four Belgians (Vandebosch, Van Cleemput, Pabian and Poels), three Spanish (Ortega-Ruiz, Casas and Garaigordobil) and one Czech (Wright), while two authors are of Asian origin (Heiman and Olenik-Shemesh). It is also necessary to highlight that in this ranking of most productive authors, 60% have contributed to research of this topic in 2019 (Vandebosch, Ortega-Ruiz, Wright, Casas, Garaigordobil and Pabian).

In addition, Table 5 indicates the most relevant keywords associated with the ten most prolific authors on the socioeconomic factors associated with CB, during 2004–2019 period. The keywords related to the works of the main authors are varied and are mainly linked to four groups. The first group is associated with the period of biological, psychological, sexual, and social development (adolescent, child, adolescence). The second group is related to harassment, inconvenience, or attacks that the person receives (computer crime, cybervictimization, cyberaggression, crime victim, cyber aggression, victim). The third is associated to the virtual space where she/he receive it (Internet). And, the fourth group associate terms related to the study and analysis of people’s behaviour and mental processes (major clinical study, psychology, loneliness, social support).

As noted, the most prominent term is “adolescent” which, as indicated by the WHO, is the period of preparation for adulthood during which certain and important experiences of development of the person take place [139].

**Figure 7:**
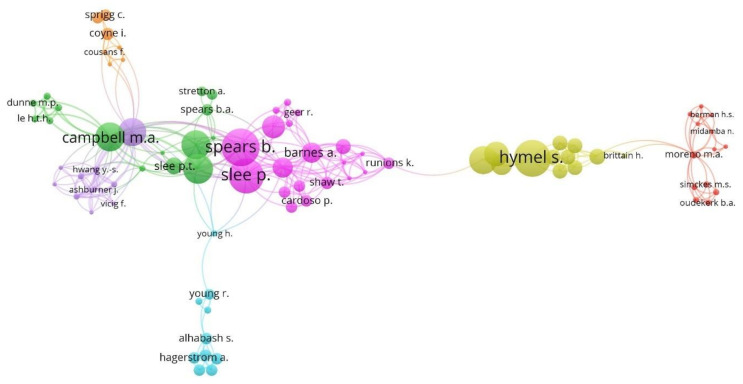
Authors network based on co-authorship analysis (2004–2019).

Figure 7 displays the cooperation map between the authors, based on co-authorship analysis, who have published globally on the socioeconomic factors associated with CB. Likewise, the colour of the cluster is related with the group of authors in the publication of articles, while the diameter of the circle indicates the number of articles by the author. Likewise, the authors in this research topic are associated in seven groups.

Cluster 1 (rose) groups 22.99%, and presents the collaboration between Barnes, Cardoso, Cross, Green, Lester, Slee, and Water, among others. Cluster 2 (green) brings together 17.24% of authors, and includes the collaboration by, among others, Butler, Campbell, Dunne, Kifts, Spears, Tran, and Whiteford. Cluster 3 (red) groups 14.94% and is composed by authors like Donlin, Moreno, Rivara, Selkie, and Simonetti, among others. Cluster 4 (yellow) links 13.79% and presents the collaboration of, among others, Brittain, Duku, Law, Short and Trinh. Cluster 5 (violet) links 11.49% of the authors and presents the cooperation of, among others, Bobir, Campbell, Tones and Vicig, F. Cluster 6 (blue) links 11.49% and describes the collaboration of, among others, Miles, Young, Richards and Rifon. Finally, cluster 7 (orange) groups 8.05% and includes the collaboration between, among others, Cousans, Coyne, Garland and Sprigg.

In the literature review, one of the main keywords that the authors constantly associate throughout the research and associate it with the CB is the term “adolescent”. Considering the grouping of the clusters, it turns out to be the keyword of the third group and the keyword most studied by the most productive authors (Table 5). Various researches acknowledge that the adolescent is going through a stage with profound physical, emotional and psychological changes, that added to the technological advances and the increase of virtual connectivity it will be for the harassers the perfect and most effective means of attacking their victims. ICT does not pose any difficulties to them, they avoid face-to-face confrontation, the extent of the damage they get is considerable in proportion to the action they execute. Throughout its history, humanity has never had so many ICTs at its disposal as at present, technologies that duplicate at high speed thanks to digitization, making it transmedia, that is, the observation of media content in different devices, multiply the ways in which we can interact people with information [140].

Regarding the first cluster whose main keyword is “psychology”, it is not observed that the most productive authors have placed it as a keyword, although among all scientific production it continues to appear as the most used keyword.

Cybervictimization is another main keyword that represents the greatest influence on research. It belongs to cluster 4, where “victim” is the keyword that represents it. In this sense, CB appears in cluster 5, as the central theme. Various investigations relate it to “computer crime” which is one of the main keywords, inferring that the authors with the greatest scientific production investigate this question. Likewise, the co-occurrence or proximity of the CB term is what allows us to understand the principle of relationships with the rest of the keywords.

In relation to cluster 7, where the central keyword is “crime victim”, it evidences the incorporation of other terms such as “socioeconomics factors”, “violence”, “socioeconomic”, or “well-being”. In this sense, authors from European Universities in Belgium and Spain, Vandebosch [141] and Garaigordobil [142], respectively, are the ones that stand out.

On the other hand, the 1128 articles on socioeconomic implications on CB research have been written in 2155 international affiliations. Table 5 displays the ten most prolific institutions in this research topic. Therefore, the top fine productive were the Universiteit Antwerpen (Belgium), University of Cordoba (Spain), Queensland University of Technology (Australia), University of Toronto (Canada) and Masaryk University (Czech Republic). In addition, this ranking highlight that six are of European origin, three of North American origin and two Australian. Among the six European, four are Spanish (University of Cordoba, University of Seville, University of the Basque Country, and International University of La Rioja). It is also noteworthy that all the institutions in this classification have published in the last year examined, 2019, confirming the interest in this topic.

Table 6 also shows the main keywords of the most productive institutions in the investigation of the socioeconomic implications of CB in an educational context, in the period 2004–2019. The keywords used by these ten most productive institutions are grouped into three groups. Consequently, the first group is associated with the period of biological, psychological, sexual, and social development, and with the training and learning stage (adolescent, child, adolescence, youth, student). The second group is related to the harassment or attacks that the person receives (cyber victimization, crime victim, computer crime). While, the third group associates terms related to the study and analysis of people’s behaviour and mental processes (mental health, major clinical study, psychology, depression).

**Figure 8:**
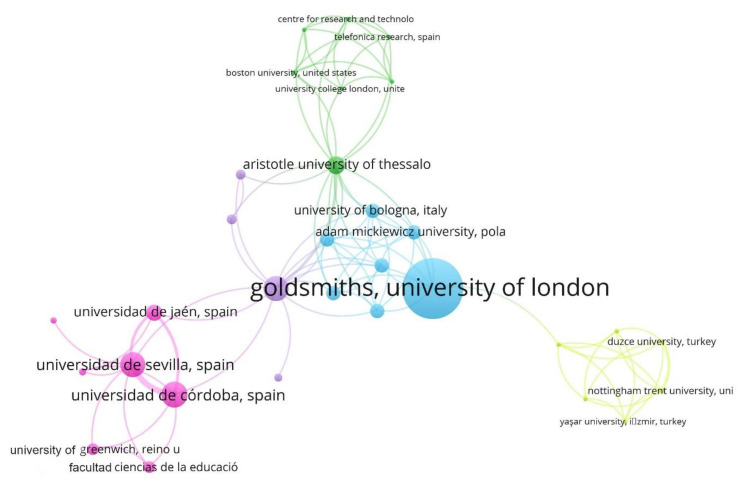
Affiliations network based on co-authorship analysis (2004–2019).

Figure 8 shows the network of institutions based on the co-authorship analysis. The VOSviewer software tool associates them into five groups. Cluster 1 (blue) groups 22.58% of the institutions, among which stand out the University of Bologna, University of Wroclaw, University Adam Mickiewicz of Poznań, or Goldsmith, University of London. Cluster 2 (green) joins 22.58%, among others, to Aristotle University of Thessaloniki, Boston University, Telefonica Research and Development or University College London. Cluster 3 (pink) associates 22.58%, highlighting the University of Jaen, University of Strathclyde, or University of Greenwich. Cluster 4 (yellow) groups 19.35% of the institutions, among them, Düzce Üniversitesi, University of Luxembourg, or Western Sydney University. Finally, cluster 5 (violet) associates 12.90%, among others, University of Southern California, University of Aveiro, or University of Bremen. It is noteworthy that the departments of various universities, such as the Spanish universities in Seville or Cordoba, are associated with different clusters.

The Spanish universities (Universities of Cordoba, Basque Country, Seville, and La Rioja) carry out their research according to the line centred on the “adolescent”. This question could be extrapolated to the rest of the European universities, such as Belgium and Czech Republic and the North American ones, which also incorporate this as the main term. The Australian Queensland University of Technology and University of South Australia focus their studies on thematic axis whose centre term is student.

In this research topic, the 1128 articles were written in 73 different countries. Table 7 shows, mainly, the top ten countries in the field of CB. The country with the most articles on CB is the United States (27.66%), followed by Spain (14.10%). Then followed by the United Kingdom (8.69%), Australia (7.09%), Canada (6.03%), and Turkey (3.99%). The rest of the countries did not exceed 3% of the total contributions. It is noteworthy that all the countries in this ranking have published their last article in 2019, that is, the last year of the period analysed, indicating the interest in this topic, in the same way as happens with institutions.

Furthermore, Table 7 also presents the three main keywords to the most productive countries in this research topic. The most used keyword in the articles is “adolescent”. In this sense, the keywords used by these ten countries are associated in four different groups. Hence, the first group is associated with the period of biological, psychological, sexual, and social development, and with the training and learning stage (adolescent, child, adolescence). The second group is related to harassment or attacks that the person receives (cyber victimization, crime victim). The third group is related to the digital environment (Internet, social media, social networking (online)). Finally, the fourth group of keywords is associated with the study and analysis of people’s behaviour and mental processes (psychology, major clinical study).

**Figure 9:**
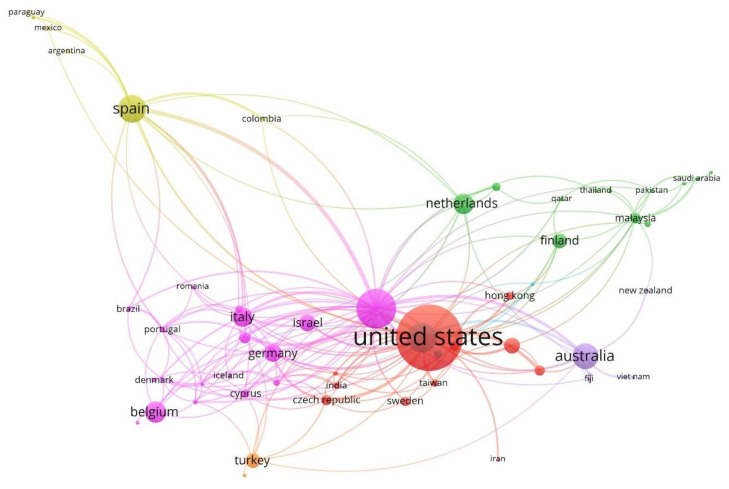
Countries network based on co-authorship analysis (2004–2019).

Figure 9 displays the collaboration network between the countries in the writing of articles on CB in a context global, based on co-authorship analysis. Once the software tool, VOSviewer, was applied to the article sample, the countries have been associated in seven clusters.

Therefore, pink cluster is the group with the most associated countries and incorporates 32.30% of the total. It is led by the United Kingdom and is mainly associated with European countries, such as Austria, Belgium, Cyprus, Denmark, Germany, Greece, Hungary, Iceland, Portugal, Romania, or Norway, and others such as Brazil and South Africa. The green cluster (22.03%) is led by the Netherlands and shares articles with Bahrain, Egypt, Finland, Iraq, Ireland, Malaysia, Nigeria, Pakistan, Qatar, and Saudi Arabia. The red cluster (18.64%) is led, in this case, by the United States, and collaborates in the production of documents on the study’s research topic, with Czech Republic, Hong Kong, India, Iran, China, Japan, Singapore, South Korea, Sweden or Taiwan. The yellow cluster (10.17%) is led by Spain and forms its network, mainly, with Latin American countries such as Argentina, Chile, Colombia, Mexico, or Paraguay. The violet cluster (8.47%) is headed by Australia, and incorporates into its network to Fiji, New Zealand, or Vietnam. The blue cluster (5.08%) is led by Canada and cooperates with France or United Arab Emirates. Finally, the orange cluster 6 (3.39%) is led by Turkey and cooperates with Ukraine.

In the development of CB research during the period analysed, 2004–2019, collaboration based on scientific activity between countries is not associated with cultural, economic, political, or legal aspects. There is unanimity in the use of the term “adolescent” by European countries (the United Kingdom, Germany, Italy, Belgium, and Spain). For their part, the United States, Australia, and Canada focus their interest mainly on the same concept. The Asian countries of Turkey and Malaysia incorporate “internet” and “social media” as main thematic axes.

In this way, as theoretical-practical implications of this research, it can be indicated that the analysis of scientific production and of the actors that stimulate CB research in an educational context, during the period 2004–2019, supposes a greater and better identification of the lines of research, in addition to their evolution and transformation. Innovation in this research field has been identified based on the composition of the clusters of authors, institutions, countries and keywords, and the intensity of the relationships that develop in them [143]. Thereby, the results obtained are a complement to the knowledge on CB in the educational context and allow the relationship between science and technique to be established, and to inform the decision-making process.

## 5. Conclusions

This work has analysed the main trends in global research on the socioeconomic factors associated with CB, in the period that begins with the publication of the first article on this research topic (2004) until the last full year (2019). For this end, the bibliometric method has been applied to a sample composed of 1128 scientific articles selected from the Elsevier Scopus database. This analysis has made it possible to obtain the evolution of scientific production, and to identify the main thematic areas, journals, authors, institutions, and countries that contribute to global research on socioeconomic aspects with implications for CB.

The volume of scientific articles has increased remarkably during these 16 years, particularly in the last five years, where 818 documents have been published, which represents 72.52% of the total contributions on the subject of social and economic aspects that affect the CB.

This study has also identified the most influential areas of knowledge: Social Sciences, Psychology, Medicine and Computer Science, which confirms the relevance of this research topic and the interest for a wide sector of the international scientific community.

Likewise, seven lines or thematic axes of research have been examined, associated, on the one hand, with the central axis of the research, that is, with CB, and, on the other, with psychology, the student, the school, the adolescent, to the victim of this type of virtual aggression, and to the term crime victim. It is a dynamic research topic that has evolved in its thematic axes during these 16 years. Since 2019, the interest in the investigation of the socioeconomic aspects that intervene and impact in the CB is confirmed due to the social nature of the phenomenon, such as race, gender, income level of the adolescent’s family, or the social environment.

Consequently, this study demonstrates the interest it arouses in scientific activity at the international level from different thematic areas, to analyse all its circumstances, highlighting, ultimately, the social and economic factors that affect this type of harassment.

This research has some limitations, which may be the basis for future research. They highlight the bibliometric methodology, which is a method that does not consider the qualitative aspects of the data. Another limitation is determining the relevance of the authors, institutions, and countries, since in the scientific field there is a paradox that some publish few articles, but they are very relevant in a certain subject area. Therefore, to overcome these limitations, in research work, bibliometric analysis must be completed with other qualitative and quantitative methodologies. On the other hand, the study could be expanded with publications collected in other databases.

Future research lines include those that will make efforts to (i) analyse the psychosocial impacts of cybervictimization and the barriers to seeking social support; (ii) carry out socio-economic analyses of the decision-making process that underlies the spectators who help victims of CB, or (iii) study the risk factors of CB and its association with perceived health in certain contexts and regions.

In conclusion, the growing interest in research on CB in the last 16 years and, recently, in the study of social and economic factors that have an impact on this type of behaviour has been observed. The relevance of this topic has been demonstrated by authors, institutions, and countries, so that the global increase in the number of contributions and the breadth of thematic axes supports the interest of the international scientific and academic community.

## Figures and Tables

**Table 1:** Key documents examined to formulate objective the research topic.

Year	Article Title [Reference]	Author(s)	Journal
2007	Prevalence and Predictors of Internet Bullying [25]	Williams, K. R.; Guerra, N. G.	*Journal of Adolescent Health*
2010	Cyberbullying: Predicting Victimisation and Perpetration. Children & Society [26]	Walrave, M.; Heirman, W.	*Children & Society*
2010	Cyber Bullying: Bullying in the Digital Age [27]	Besag, V.	*Child and Adolescent Mental Health*
2012	Is Adolescent Bullying an Evolutionary Adaptation? [28]	Volk, A. A.; Camilleri, J. A.; Dane, A. V.; Marini, Z. A.	*Aggressive Behavior*
2013	Cyber Bullying and Physical Bullying in Adolescent Suicide: The Role of Violent Behavior and Substance Use [29]	Litwiller, B. J.; Brausch, A. M.	*Journal of Youth and Adolescence*
2013	Cyberbullying in those at clinical high risk for psychosis [30]	Magaud, E.; Nyman, K.; Addington, J.	*Early Intervention in Psychiatry*
2014	Cyberbullying [31]	Bayraktar, F.; Machackova, H.; Dedkova, L.; Cerna, A.; Ševčíková, A.	*Journal of Interpersonal Violence*
2014	Socioeconomic Status and Bullying: A Meta-Analysis [32]	Tippett, N.; Wolke, D.	*American Journal of Public Health*
2015	How do victims react to cyberbullying on social networking sites? The influence of previous cyberbullying victimization experiences [33]	Cao, B.; Lin, W.-Y.	*Computers in Human Behavior*
2015	Systematic review of theoretical studies on bullying and cyberbullying: Facts, knowledge, prevention, and intervention [34]	Zych, I.; Ortega-Ruiz, R.; Del Rey, R.	*Aggression and Violent Behavior*
2017	Bullying victims’ perceptions of classroom interaction [35]	Havik, T.	*School Effectiveness and School Improvement*
2017	Cyberbullying Among Adolescents: The Influence of Different Modes of Inquiry [36]	Brochado, S.; Fraga, S.; Soares, S.; Ramos, E.; Barros, H.	*Journal of Interpersonal Violence*
2019	Are cyberbullying intervention and prevention programs effective? A systematic and meta-analytical review [37]	Gaffney, H.; Farrington, D. P.; Espelage, D. L.; Ttofi, M. M.	*Aggression and Violent Behavior*
2019	How Does Individualism-Collectivism Relate to Bullying Victimisation? [38]	Smith, P. K.; Robinson, S.	*International Journal of Bullying Prevention*
2019	Cyberbullying Victimization in WhatsApp Classmate Groups among Israeli Elementary, Middle, and High School Students [39]	Aizenkot, D.; Kashy-Rosenbaum, G.	*Journal of Interpersonal Violence*

**Table 2:** Types of bullying and cyberbullying.

Bullying	Cyberbullying
**Physical** Harm the person and/or his/her property.	**Happy beating** Record a physical assault on a person and spread it on social networks.
**Verbal** Insulting or slandering the victim.	**Electronic insults** In public chats or private e-mails.
**Social** Isolate the victim from the group.	**Harassment** Sending offensive messages on social networks.
	**Denigration** Repeatedly broadcasting offensive messages/photos on social networks.
	**Disclosure and hoax** Filtering of the victim’s compromised information on social networks.
	**Cyber pursuit:** Repeatedly sending threatening messages.
**Psychological** Damaging the victim’s self-esteem and generating insecurity and fear.	**Impersonation:** Usurpation of the victim’s identity on social networks.

**Table 3:** Most cited articles (2004–2019).

Year	Tittle [Reference]	Author(s)	Journal	Total Citations	Subject Area
2008	Cyberbullying: Its nature and impact in secondary school pupils [107]	Smith, P.K., Mahdavi, J., Carvalho, M., (…), Russell, S., Tippett, N.	Journal of Child Psychology and Psychiatry and Allied Disciplines	1316	Medicine
2009	School Bullying Among Adolescents in the United States: Physical, Verbal, Relational, and Cyber [108]	Wang, J., Iannotti, R.J., Nansel, T.R.	Journal of Adolescent Health	907	Medicine
2006	Bullies Move Beyond the Schoolyard: A Preliminary Look at Cyberbullying [109]	Patchin, J.W., Hinduja, S.	Youth Violence and Juvenile Justice	749	Social Sciences
2008	Cyberbullying: Another main type of bullying?: Personality and Social Sciences [110]	Slonje, R., Smith, P.K.	Scandinavian Journal of Psychology	737	Arts and Humanities
2007	Electronic Bullying Among Middle School Students [111]	Kowalski, R.M., Limber, S.P.	Journal of Adolescent Health	695	Medicine
2008	Extending the school grounds?—Bullying experiences in cyberspace [112]	Juvonen, J., Gross, E.F.	Journal of School Health	648	Arts and Humanities
2013	Psychological, physical, and academic correlates of cyberbullying and traditional bullying [113]	Kowalski, R.M., Limber, S.P.	Journal of Adolescent Health	398	Social Sciences
2008	Cyberbullying: Youngsters’ experiences and parental perception [114]	Dehue, F., Bolman, C., Völlink, T.	Cyberpsychology and Behavior	355	Medicine
2010	Psychosocial risk factors associated with cyberbullying among adolescents: A population-based study [115]	Sourander, A., Klomek, A.B., Ikonen, M., (…), Ristkari, T., Helenius, H.	Archives of General Psychiatry	333	Medicine
2010	Cyberbullying in adolescents: Modalities and aggressors’ profile [116]	Calvete, E., Orue, I., Estévez, A., Villardón, L., Padilla, P.	Computers in Human Behavior	282	Arts and Humanities

**Table 4:** Main keywords by occurrences and total link strength (2004–2019).

	Keyword	Cluster Colour (see Figure 5)	Occurrences	Total Link Strength
1	cyberbullying	violet	666	1929
2	bullying	pink	408	1778
3	adolescent	red	340	1899
4	Internet	red	256	1227
5	child	red	203	1208
6	cyber bullying	violet	177	513
7	psychology	pink	159	1083
8	social media	violet	154	558
9	major clinical study	yellow	149	881
10	crime victim	orange	138	1042
11	computer crime	violet	116	375
12	student	green	116	781
13	victim	yellow	116	677
14	adolescent behavior	red	113	756
15	adolescence	green	95	427
16	aggression	red	89	481
17	social networking (online)	violet	83	247
18	school	blue	78	530
19	education	green	71	334
20	mental health	pink	70	1662

**Table 5:** Most productive authors and main keywords (2004–2019).

R	Author	Affiliation	Country	1A	LA	Main Keywords		
						1	2	3
1	Vandebosch	Universiteit Antwerpen	Belgium	2008	2019	Adolescent	Child	Computer Crime
2	Ortega-Ruiz	University of Cordoba	Spain	2012	2019	Adolescent	Cybervictimization	Major Clinical Study
3	Wright	Masaryk University	Czech Republic	2013	2019	Adolescent	Cybervictimization	Cyber Aggression
4	Van Cleemput	Universiteit Antwerpen	Belgium	2008	2018	Adolescent	Computer Crime	Child
5	Casas	University of Cordoba	Spain	2015	2019	Cybervictimization	Adolescence	Cyberaggression
6	Garaigordobi	University of the Basque Country	Spain	2014	2019	Adolescence	Crime Victim	Psychology
7	Heiman	Open University of Israel	Israel	2013	2018	Adolescent	Social Support	Loneliness
8	Olenik-Shemesh	Open University of Israel	Israel	2012	2018	Adolescent	Social Support	Loneliness
9	Pabian	Universiteit Antwerpen	Belgium	2012	2019	Adolescent	Child	Internet
10	Poels	Universiteit Antwerpen	Belgium	2012	2018	Computer Crime	Victim	Adolescent

R: position in the ranking; N: number of articles by country; %: percentage of total articles; 1A: First article in research topic; LA: Last article in research topic.

**Table 6:** Most productive affiliations and main keywords (2004–2019).

R	Institution	N	%	Country	First Article	Last Article	Main Keywords		
							1	2	3
1	Universiteit Antwerpen	28	2.48	Belgium	2005	2019	Adolescent	Child	Computer Crime
2	University of Cordoba	27	2.39	Spain	2012	2019	Adolescent	Child	Student
3	Queensland University of Technology	17	1.51	Australia	2009	2019	Student	Mental Health	Adolescent
4	University of Toronto	16	1.42	Canada	2009	2019	Adolescent	Major Clinical Study	Mental Health
5	Masaryk University	16	1.42	Czech Republic	2011	2019	Adolescent	Child	Crime Victim
6	University of Seville	16	1.42	Spain	2012	2019	Adolescence	Adolescent	Child
7	University of the Basque Country	15	1.33	Spain	2013	2019	Adolescent	Adolescence	Psychology
8	University of South Australia	13	1.15	Australia	2009	2019	Mental Health	Student	Youth
9	International University of La Rioja	13	1.15	Spain	2018	2019	Adolescent	Child	Adolescence
10	Pennsylvania State University	12	1.06	USA	2016	2019	Adolescent	Cyber Victimization	Depression

R: position in the ranking; N: number of articles by country; %: percentage of total articles.

**Table 7:** Most productive countries and main keywords (2004–2019).

R	Country	N	%	First Article	Last Article	Main Keywords		
						1	2	3
1	USA	312	27.66	2006	2019	Adolescent	Internet	Psychology
2	Spain	159	14.10	2010	2019	Adolescent	Adolescence	Internet
3	UK	98	8.69	2015	2019	Adolescent	Internet	Child
4	Australia	80	7.09	2009	2019	Adolescent	Social Media	Crime Victim
5	Canada	68	6.03	2004	2019	Adolescent	Child	Social Media
6	Turkey	45	3.99	2009	2019	Internet	Adolescent	Major Clinical Study
7	Belgium	33	2.93	2008	2019	Adolescent	Child	Internet
8	Germany	33	2.93	2009	2019	Adolescent	Internet	Crime Victim
9	Italy	29	2.57	2015	2019	Adolescent	Internet	Child
10	Malaysia	28	2.48	2015	2019	Social Media	Social Networking (online)	Computer Crime

R: position in the ranking; N: number of articles by country; %: percentage of total articles.
